# The relationship between common mental disorders (CMDs), food insecurity and domestic violence in pregnant women during the COVID-19 lockdown in Cape Town, South Africa

**DOI:** 10.1007/s00127-021-02140-7

**Published:** 2021-07-19

**Authors:** Zulfa Abrahams, Sonet Boisits, Marguerite Schneider, Martin Prince, Crick Lund

**Affiliations:** 1 Department of Psychiatry and Mental Health, Alan J Flisher Centre for Public Mental Health, University of Cape Town, Building B, 46 Sawkins Road, Rondebosch, Cape Town, South Africa; 2 Health Service and Population Research Department, Centre for Global Mental Health, Institute of Psychiatry, Psychology and Neuroscience, King’s Global Health Institute, King’s College London, London, UK

**Keywords:** COVID-19, Maternal, Mental health, Depression, Food insecurity

## Abstract

**Purpose:**

We aimed to explore the relationship between common mental disorders (CMDs), food insecurity and experiences of domestic violence among pregnant women attending public sector midwife obstetric units and basic antenatal care clinics in Cape Town during the COVID-19 lockdown.

**Methods:**

Perinatal women, attending 14 healthcare facilities in Cape Town, were enrolled in the study during baseline data collection before the COVID-19 lockdown. During the lockdown period, fieldworkers telephonically contacted the perinatal women who were enrolled in the study and had provided contact details. The following data were collected from those who consented to the study: socio-demographic information, mental health assessment, food insecurity status and experiences of domestic violence. Poisson regression was used to model the associations of a number of risk factors with the occurrence of CMDs.

**Results:**

Of the 2149 women enrolled in the ASSET study, 885 consented to telephonic interviews. We found that 12.5% of women had probable CMDs and 43% were severely food insecure. Psychological distress increased significantly during the lockdown period, compared to before the COVID-19 outbreak. Using multivariate Poisson regression modelling, we showed that the risk of CMDs was increased in women who were severely food insecure or who experienced psychological or sexual abuse.

**Conclusions:**

This study provides evidence of the effect of the COVID-19 lockdown on the mental health status of perinatal women living in low-resource settings in Cape Town and highlights how a crisis such as the COVID-19 lockdown amplifies the psycho-social risk factors associated with CMDs in perinatal women.

**Supplementary Information:**

The online version contains supplementary material available at 10.1007/s00127-021-02140-7.

## Introduction

Common mental disorders (CMDs) such as depression and anxiety are highly prevalent during the perinatal period, especially in low- and middle-income countries where approximately 19% of perinatal women develop depression [1], and approximately 34% develop anxiety [2]. In South Africa the prevalence is particularly high, with several studies reporting that one in every three pregnant women develop symptoms of depression [3–7], and one in every four pregnant women develop symptoms of anxiety [3, 8]. However, studies have also shown that the majority of women who experience mild to moderate symptoms of depression during their first and second trimester of pregnancy, show an improvement in symptoms during the remainder of the perinatal period, even without intervention [7, 9–11]. Studies in Cape Town, South Africa have identified several psycho-social risk factors for CMDs which include food insecurity, intimate partner violence, and lack of social support [4, 7, 12].

The Coronavirus (COVID-19) outbreak was declared a global pandemic by the World Health Organisation on 11 March 2020 [13]. A few days later the President of South Africa announced that urgent and drastic measures were needed to manage the disease [14]. This included a travel ban, increased screening and testing and the closure of all schools. Two weeks later, a 35-day national lockdown came into effect [15, 16]. This initial lockdown period became known as Alert Level-5 and required South Africans to stay home except for essential purposes. All non-essential activities were suspended until the end of April 2020, including the sale of alcohol [17]. During May 2020, South Africa moved to Alert Level-4, which resulted in a slight easing of restrictions. However, South Africans remained confined to their place of residence, except for those performing essential services [18]. On 1 June 2020, Alert Level-3 came into effect, further easing restrictions, which included unbanning the sale of alcohol and the opening of schools and some places of employment [19].

According to the World Health Organisation [20], experiencing fear, worry and distress is an understandable and normal response in the context of the COVID-19 pandemic. The South African Depression and Anxiety Group (SADAG) reported that the COVID-19 lockdown in South Africa resulted in an increase in the number of calls from people feeling overwhelmed, anxious, worried and depressed. In an online survey completed between 2 and 15 April 2020, SADAG found that 65% of respondents were feeling stressed during the lockdown [21]. Pregnant women and mothers of young children are especially vulnerable to distress during the lockdown [7, 22]. Stressors highlighted include (1) the effects of social distancing and isolation, such as a lack of social support from family and friends, (2) financial difficulties, (3) the increased risk of intimate partner violence as women are forced to remain in close proximity to the perpetrator, (4) reduced antenatal and postnatal check-ups, and (5) partners’ inability to participate in the birth [23, 24].

South Africa is a country characterised by high levels of inequality and poverty [25]. The majority of South Africans (55%) live below the upper bound poverty line [26], with many living in overcrowded shacks with intergenerational families. Intimate partner violence rates are high and is thought to be associated with poverty and food insecurity [27]. The lockdown has been especially difficult for the already vulnerable groups living in poverty, as it has resulted in increased levels of unemployment, food insecurity [28] and domestic violence [29]. However, no studies have reported on effect of the COVID-19 crisis on the mental health of perinatal women in South Africa. As unemployment, food insecurity and domestic abuse is associated with CMDs, this study aims to explore the relationship between CMDs, food insecurity and experiences of violence among pregnant women attending public healthcare facilities in Cape Town during the COVID-19 lockdown.

## Methods

### Setting

This quantitative study was conducted with perinatal women attending 14 randomly selected midwife obstetric units (MOUs) or basic antenatal care (BANC) clinics in the Cape Metropolitan Health District in Cape Town. MOUs and BANC clinics are public sector antenatal and obstetric care facilities managed by the Western Cape Department of Health. The facilities are free at the point of contact and attended predominately by women living in low socio-economic status communities. This study forms part of the bigger Health Systems Strengthening in sub-Saharan Africa (ASSET) study [30]—which includes a cluster randomised control trial (ISRTCN41483663) to evaluate an intervention to strengthen detection, referral and care for antenatal women with depression, anxiety and experiences of domestic violence in Cape Town.

In February and March 2020, 2149 perinatal women were enrolled in the ASSET study which occurred prior to the COVID-19 pandemic in South Africa. Data collected included a file review of their current Maternity Case Records (MCR)—the national stationery used to record all aspects of the pregnancy [31]. Information collected from the MCR included the patients’ contact details, gestational and medical history, and results of a 3-question mental health screening questionnaire used to assess psychological distress [32]. The mental health screening questionnaire is routinely administered by health professionals during patients’ first antenatal visit. MCRs of women who were pregnant (irrespective of gestation period) or had given birth in the past three months were included in the ASSET study’s baseline data collection. As a result of the COVID-19 pandemic, data collection was suspended after completion of the baseline data collection, but before the intervention could begin. During the period when face-to-face data collection was not permitted, the study was opportunistically pivoted to focus on the impact of the COVID-19 lockdown on women already enrolled in the ASSET study.

### Testing procedures

During June and July 2020 (Alert Level-3 lockdown), fieldworkers telephonically contacted the perinatal women who were enrolled in the ASSET study and had provided contact details and invited them to participate in a telephonic survey. Questionnaires were telephonically administered to perinatal women who consented to the study. Questionnaires were available in English, Afrikaans or IsiXhosa, and included (1) a socio-demographic questionnaire that was used to collect data on participants’ age, obstetric information, relationship status, income status, and effect of the COVID-19 lockdown, (2) the same 3-question mental health screening questionnaire [32] that was administered by healthcare workers at patients’ first clinic visit, (3) the Edinburgh Postnatal Depression Scale (EPDS) [33], (4) the Household Food Insecurity and Access Scale (HFIAS) [34], and (5) the short form of the Composite Abuse Scale (CAS-SF) [35].

The EPDS is commonly used as a screening tool in research settings and has been validated against the Diagnostic and Statistical Manual (DSM-IV) [36, 37] for depression and anxiety in a sample of postnatal women in South Africa [33, 38] with a cut-off of ≥ 13 indicating a probable CMD. The questionnaire consists of 10 items with a seven-day recall period. The 3-question mental health screening questionnaire uses a two-week recall period and has been validated against the EPDS [32]. Both the 3-question mental health screening questionnaire and the EPDS asks about suicidality. Using a cut-point of ≥ 2, this screening tool is able to identify perinatal women with psychological distress (sensitivity = 85.7%; specificity = 92.9%). The HFIAS was used to assess household food insecurity and hunger [34]. This 9-item scale measures the household’s frequency of running out of food or eating inadequate amounts of food during the past 30 days. The CAS-SF [35] is a 15-item instrument that captures women’s self-reported experience of physical, sexual and psychological abuse during the past 12 months.

### Data analysis

Data analysis was carried out using STATA/SE statistical software package version 15.1 (StataCorp., College Station, TX, USA). All participants with incomplete data were excluded from the analysis. Variables were described using frequency and percentages, and associations measured using Chi-square tests. As the mental health screening questionnaire was administered in-person by healthcare workers, at patients’ first clinic visit, and telephonically by trained fieldworkers during the lockdown, the Bland–Altman analysis was used to measure agreement between the two time-points [39, 40].

Psychological distress was calculated using the mental health screening questionnaire. Women who scored two or more on the questionnaire were classified as experiencing psychological distress. Univariate logistic regression analysis, using psychological distress as the outcome variable, was used to model the associations of several risk factors with the binary outcome variable. Household food security status was calculated [41] using the HFIAS to categorise households into four levels of household food insecurity experienced during the past 30 days: food secure, mildly food insecure, moderately food insecure and severely food insecure. The CAS-SF was used to categorise experiences of domestic violence during the past 12 months into physical, sexual and psychological abuse [35].

Poisson regression analysis was used to prevent overestimation of the risk [42]. Variables with a *p* value of < 0.2 in the univariate Poisson regression were entered in the multivariate model to generate adjusted risk ratios [43, 44].

### Ethical approval

Ethical approval for the study was obtained from the Human Research Ethics Committee at the Faculty of Health Sciences, University of Cape Town (Ref No: 139/2018) and the Psychiatry, Nursing and Midwifery Research Ethics Subcommittee at Kings College London (Ref No: 17/18–7807). In addition, the Western Cape Department of Health approved the use of the research sites (Ref No: WC_201807_008). Consent forms were available in English, Afrikaans and isiXhosa. Participants provided informed consent after the procedure had been verbally explained to them. All participants were informed that they were free to withdraw from the study at any time without consequences. No financial incentives were provided for participating in the study.

## Results

Data from MCRs were available for 2149 perinatal women (Supplementary Table). More than half the women (*n* = 1248; 58%) who were initially enrolled in the ASSET study, were not contactable due to missing or incorrect contact details. An additional 16 women declined to participate when contacted. In total, 1264 women were lost-to-follow-up (response rate = 41.2%). Gravidity (*p* = 0.672), pregnancy status (*p* = 0.097) and the results of a routinely administered mental health screening questionnaire (*p* = 0.199) were similar for those included in the study and those who were lost to follow-up. Significantly more young women aged 15–24 years were lost-to-follow-up (*p* = 0.047).

Bivariate associations between CMD (measured using the EPDS) and the socio-demographic and psychological characteristics of participants are presented in Table 1. The study sample consisted of 885 participants, of whom 110 (12.4%) were classified as having a probable CMD and 775 (87.6%) were not. Almost half the women were severely food insecure (*n* = 378), while more than half were unemployed (*n* = 475). The proportion of women with CMD significantly increased with increasing number of pregnancies (*p* = 0.017) and severity of food insecurity (*p* < 0.001). Significantly fewer women who had experienced their first pregnancy (20.9 vs. 30.2%; *p* = 0.045) and had already given birth at follow-up had a probable CMD (63.6 vs. 74.1%; *p* < 0.022).

**Table 1:** Bivariate associations between socio-demographic and psycho-social risk factors and probable CMD

	With CMD* (*n* = 110; 12.4%) *n* (%)	Without CMD (*n* = 775; 87.6%) *n* (%)	Chi-square tests of independence	Jonckheere-Terpstra test for trend
Age				
15–24 years	29 (26.4)	262 (33.9)	*χ*^2^(1) = 2.498; *p* = 0.114	*J* = 39,193; *p* = 0.378
25–29 years	40 (36.4)	226 (29.3)	*χ*^2^(1) = 2.297; *p* = 0.130	
30–35 years	28 (25.4)	187 (24.2)	*χ*^2^(1) = 0.079; *p* = 0.778	
> 35 years	13 (11.8)	97 (12.6)	*χ*^2^(1) = 0.049; *p* = 0.825	
Gravidity				
1st pregnancy	23 (20.9)	232 (30.2)	*χ*^2^(1) = 4.007; *p* = 0.045	*J* = 47,353; *p* = 0.017
2–4 pregnancies	74 (67.3)	483 (62.8)	*χ* (1) = 0.826; *p* = 0.363	
> 4 pregnancies	13 (11.8)	54 (7.0)	*χ*^2^(1) = 3.144; *p* = 0.076	
Number of other children				
0 children	36 (33.0)	294 (38.2)	*χ*^2^(1) = 1.102; *p* = 0.294	*J* = 44,602; *p* = 0.226
1–2 children	59 (54.1)	396 (51.5)	*χ*^2^(1) = 0.265; *p* = 0.607	
3–4 children	11 (10.1)	72 (9.4)	*χ*^2^(1) = 0.059; *p* = 0.808	
> 4 children	3 (2.8)	7 (0.9)	*χ*^2^(1) = 2.877; *p* = 0.090	
Pregnancy status				
Postnatal	70 (63.6)	568 (74.1)	*χ*^2^(1) = 5.267; *p* = 0.022	
Pregnant	34 (30.9)	192 (25.0)	*χ*^2^(1) = 1.737; *p* = 0.188	
Miscarriage/ Stillbirth	6 (5.5)	7 (0.9)	*χ*^2^(1) = 13.590; *p* < 0.001	
Unplanned pregnancy	75 (69.4)	477 (62.6)	*χ*^2^(1) = 0.912; *p* = 0.167	
No partner	20 (18.4)	86 (11.4)	*χ*^2^(1) = 4.355; *p* = 0.037	
Psychological distress at first clinic visit**	5 (9.1)	12 (2.5)	*χ*^2^(1) = 7.168; *p* = 0.007	
Anxiety/worry about getting infected***	100 (90.9)	640 (83.1)	*χ*^2^(1) = 4.368; *p* = 0.037	
Employment status prior to the lockdown				
Employed	44 (40.0)	304 (39.5)	*χ*^2^(1) = 0.011; *p* = 0.917	
Unemployed	58 (52.7)	417 (54.1)	*χ*^2^(1) = 0.079; *p* = 0.799	
Student	8 (7.3)	49 (6.4)	*χ*^2^(1) = 0.131; *p* = 0.717	
Increased crime in community****	37 (33.6)	172 (22.5)	*χ*^2^(1) = 6.621; *p* = 0.010	
Increased domestic violence***	5 (4.6)	23 (3.0)	*χ*^2^(1) = 0.783; *p* = 0.376	
Decreased income***	80 (72.7)	496 (64.6)	*χ*^2^(1) = 2.828; *p* = 0.093	
Decreased amount of food in household***	98 (89.1)	551 (71.6)	*χ*^2^(1) = 15.282; *p* < 0.001	
Food security status***				
Food secure	7 (6.5)	167 (21.7)	*χ*^2^(1) = 13.858; *p* < 0.001	*J* = 54,498; *p* < 0.001
Mildly food insecure	6 (5.6)	75 (9.8)	*χ*^2^(1) = 1.999; *p* = 0.157	
Moderately food insecure	22 (20.4)	221 (28.8)	*χ*^2^(1) = 3.337; *p* = 0.068	
Severely food insecure	73 (67.6)	305 (39.7)	*χ*^2^(1) = 30.000; *p* < 0.001	
Experiences of abuse***				
Psychological abuse	36 (34.3)	101 (13.4)	*χ*^2^(1) = 30.005; *p* < 0.001	
Physical abuse	33 (31.1)	88 (11.7)	*χ*^2^(1) = 29.112; *p* < 0.001	
Sexual abuse	8 (7.5)	6 (0.8)	*χ*^2^(1) = 26.667; *p* < 0.001	

*Probable common mental disorder (CMD), scored ≥ 13 on the Edinburgh Postnatal Depression Scale (EPDS)

**Scored ≥ 2 on routinely administered mental health screening questionnaire

***During the Covid-19 lockdown

Significantly more women with a probable CMD had experienced a previous miscarriage or stillbirth (5.5 vs. 0.9%; *p* < 0.001), did not have a partner (18.4 vs. 11.4%; *p* = 0.037), experienced psychological distress at the first clinic visit (9.1 vs. 2.5%; *p* = 0.007), experienced anxiety about being infected with the COVID-19 virus (90.9 vs. 83.1%; *p* = 0.037), experienced an increase in crime in their community (33.6 vs. 22.5%; *p* = 0.010) and a decrease in food available in the household (89.1 vs. 71.6%; *p* < 0.001) during the lockdown, were severely food insecure (67.6 vs. 39.7%; *p* < 0.001) and experienced psychological (34.3 vs. 13.4%; *p* < 0.001), physical (31.1 vs. 11.7%; *p* < 0.001) or sexual abuse (7.5 vs. 0.8%; *p* < 0.001), compared to women without CMD.

A comparison of the results of the routinely administered mental health screening tool is presented in Table 2. Five hundred and fifty-five participants completed the screening questionnaire at their first clinic visit and during the lockdown. During the lockdown, the proportion of women who felt unable to stop worrying or thinking too much increased more than three-fold (12–40%), while the number of women who felt down, depressed or hopeless increased by six times (5–30%). There was no change in the number or proportion of women who felt suicidal. Significantly more women experienced psychological distress during the lockdown, compared to their first clinic visit (3.1 vs. 26.2%; *p* < 0.001). Using the EPDS as the gold standard, a cut-point of ≥ 2 on the mental health screening questionnaire was able to correctly classify 88.6% of participants (sensitivity = 0.71; specificity = 0.91).

**Table 2:** Comparison of the results of the routinely administered mental health screening questionnaire measuring psychological distress before and during the lockdown (*n* = 555)

	At the 1st clinic visit *n* (%)	During the lockdown *n* (%)	Bland–Altman tests	Chi-square test
Question 1: have you on some or most days felt unable to stop worrying or thinking too much?	66 (11.9)	221 (39.8)	*r* = 0.392; *p* < 0.001	*χ*^2^(1) = 160.790; *p* < 0.001
Question 2: have you on some or most days felt down, depressed or hopeless?	27 (4.9)	168 (30.4)	*r* = 0.640; *p* < 0.001	*χ*^2^(1) = 144.048; *p* < 0.001
Question 3: have you on some or most days had thoughts and plans to harm yourself or commit suicide?	10 (1.8)	10 (1.8)	*r* = 0.00; *p* = 1.0	*χ*^2^(1) = 0.0719; *p* = 0.789
Psychological distress*	17 (3.1)	230 (26.2)	*r* = 0.720; *p* < 0.001	*χ*^2^(1) = 126.817; *p* < 0.001
Mental Health Score** [mean (± SD)]	0.18 (± 0.49)	0.72 (± 0.86)	*r* = 0.505; *p* < 0.001	*t* = 12.985; *p* < 0.001***

*Score of ≥ 2 on the mental health screening questionnaire

**Sum of questions 1–3 (min = 0; max = 3)

***t test

Univariate logistic regression of risk factors against the outcome, psychological distress at the first clinic visit and during the lockdown is presented in Table 3. Several MCRs contained incomplete mental health screening questionnaires, resulting in fewer participants with a measure of psychological distress at the first clinic visit compared to during the lockdown. Only experiences of psychological abuse were significantly associated with psychological distress at the first clinic visit [Odds ratio (OR) 3.65; 95% Confidence interval (CI) 1.26–10.55]. The odds of experiencing psychological distress during the lockdown were significantly associated with: more than four previous pregnancies (OR 1.74; 95% CI 1.04–2.93); a previous miscarriage or stillbirth (OR 9.72; 95% CI 2.65–35.66); no partner (OR 2.23; 95% CI 1.47–3.40); an increase in crime in their community (OR 2.01; 95% CI 1.43–2.80); less income during the lockdown (OR 1.68; 95% CI 1.20–2.35); less food in the household during the lockdown (OR 2.24; 95% CI 1.52–3.31); severe food insecurity during the lockdown (OR 2.39; 95% CI 1.76–3.25); psychological (OR 3.05; 95% CI 2.09–4.46) physical (OR 2.77; 95% CI 1.86–4.11) or sexual (OR 7.30; 95% CI 2.27–23.53) abuse in the past 12 months.

**Table 3:** Univariate logistic regression models: factors associated with psychological distress at the first clinic visit and during the lockdown

	Psychological distress*: 1st clinic visit (*n* = 543)	Psychological distress: lockdown (*n* = 869)
	Odds ratio (95% confidence interval)	
Age		
15–24 years	1.23 (0.71–2.16)	0.61 (0.44–0.86)
25–29 years	0.86 (0.44–1.66)	1.21 (0.88–1.68)
30–35 years	0.97 (0.50–1.89)	1.33 (0.95–1.87)
> 35 years	0.77 (0.23–2.19)	1.06 (0.68–1.66)
Gravidity		
1st pregnancy	0.63 (0.33–1.21)	0.63 (0.44–0.89)
2–4 pregnancies	1.56 (0.86–2.81)	1.23 (0.89–1.68)
> 4 pregnancies	0.86 (0.31–2.44)	1.74 (1.04–2.93)
Number of children		
0 children	0.81 (0.36–1.82)	0.61 (0.44–0.86)
1–2 children	0.72 (0.30–1.70)	1.21 (0.88–1.68)
3–4 children	0.34 (0.81–1.40)	1.33 (0.95–1.87)
> 4 children	1.76 (0.96–3.21)	1.06 (0.68–1.66)
Pregnancy status		
Post-birth	1.46 (0.82–2.63)	0.88 (0.63–1.123)
Pregnant	0.68 (0.39–1.22)	0.93 (0.66–1.31)
Miscarriage/Stillbirth		9.72 (2.65–35.66)
Unplanned pregnancy	1.42 (0.49–4.09)	1.28 (0.93–1.76)
No partner	1.16 (0.26–5.21)	2.23 (1.47–3.40)
Employment status prior to the lockdown		
Employed	0.73 (0.27–2.02)	0.81 (0.59–1.10)
Unemployed	1.33 (0.50–3.55)	1.32 (0.97–1.78)
Student	1.05 (0.13–8.17)	0.74 (0.38–1.42)
Increased crime in community**		2.01 (1.43–2.80)
Increased domestic violence**		3.96 (1.84–8.52)
Decreased income**		1.68 (1.20–2.35)
Decreased amount of food in household**		2.24 (1.52–3.31)
Food security status during the past 30 days**		
Food secure		0.33 (0.20–0.53)
Mildly food insecure		0.67 (0.38–1.19)
Moderately food insecure		0.82 (0.58–1.16)
Severely food insecure		2.39 (1.76–3.25)
Experiences of abuse during the past 12 months**		
Psychological abuse		3.05 (2.09–4.46)
Physical abuse		2.77 (1.86–4.11)
Sexual abuse		7.30 (2.27–23.53)

*Scored ≥ 2 on mental health screening questionnaire

**During the Covid-19 lockdown

The relative risk of experiencing a probable CMD is shown in Table 4. In the univariate model, having already given birth [Risk ratio (RR) = 0.66; 95% CI 0.44–0.97] was negatively associated with experiencing a probable CMD, while having had a previous miscarriage or stillbirth (RR 3.83; 95% CI 1.68–8.73), experiencing increased crime in the community (RR 1.62; 95% CI 1.09–2.40), experiencing psychological distress at the first clinic visit (RR 3.09; 95% CI 1.23–7.76), being severely food insecure (RR 2.75; 95% CI 1.84–4.11) or experiencing physical (RR 2.76; 95% CI 1.83–4.16), psychological (RR 2.75; 95% CI 1.84–4.11]) or sexual (RR 4.96; 95% CI 2.41–10.20) abuse was associated with a probable CMD.

**Table 4:** Poisson regression model: factors associated with probable CMDs

	Unadjusted model	Multivariate model*
	Risk ratio (95% confidence interval)	
1st pregnancy (ref: > 4 pregnancies)	0.65 (0.41–1.02)	0.58 (0.31–1.09)
2–4 pregnancies (ref: > 4 pregnancies)	1.19 (0.80–1.77)	
Post-birth (ref: pregnant)	0.66 (0.44–0.97)	
Miscarriage/stillbirth (ref: pregnant)	3.83 (1.68–8.73)	3.60 (1.15–11.21)
No partner	1.61 (0.99–2.62)	
Increased crime in community**	1.62 (1.09–2.40)	1.50 (0.82–2.71)
Psychological distress at first clinic visit***	3.09 (1.23–7.76)	
Mildly food insecure*** (ref: food secure)	0.58 (0.25–1.31)	
Moderately food insecure*** (ref: food secure)	0.67 (0.42–1.06)	
Severely food insecure*** (ref: food secure)	2.75 (1.84–4.11)	3.01 (1.60–5.65)
Physical abuse	2.76 (1.83–4.16)	
Psychological abuse	2.75 (1.84–4.11)	2.38 (1.25–4.54)
Sexual abuse	4.96 (2.41–10.20)	2.53 (1.03–6.24)

*Variables with a *p* value of < 0.2 in the univariate model were entered in the multivariate model

**During the Covid-19 lockdown

***Scored ≥ 2 on routine mental health screening questionnaire

In the multivariate model, women were at increased risk of a probable CMD if they had a previous miscarriage or stillbirth (RR 3.60; 95% CI 1.15–11.21), experienced severe food insecurity (RR 3.01; 95% CI 1.60–5.65), or experienced psychological abuse (RR 3.38; 95% CI 1.25–4.54) or sexual abuse (RR 2.53; 95% CI 1.03–6.24).

## Discussion

As little is known about the effects of the COVID-19 lockdown on known risk factors for perinatal depression, this study explored the relationship between the mental health status of perinatal women, and their experiences of food insecurity and domestic violence during the COVID-19 lockdown. We found that 12% of women had probable CMDs and 43% were severely food insecure. Levels of psychological distress increased significantly during the lockdown period, compared to before the COVID-19 outbreak in South Africa. While we did not find an increase in women who felt suicidal, significantly more women reported feeling anxious and depressed. Using multivariate Poisson regression modelling, we showed that the risk of CMD was almost three times greater in women who were severely food insecure, or who experienced psychological or sexual abuse. Importantly, we found strong associations between certain risk factors (having more than four pregnancies or a previous miscarriage or stillbirth, experiencing increased crime in the community, decreased income or less food in the household, severe food insecurity, or any form of abuse) and psychological distress during the COVID-19 lockdown.

Compared to other studies in South Africa [3, 4, 7, 12], our study found a relatively low prevalence of CMDs (12.5% vs. 15–40%) in pregnant women during the COVID-19 lockdown, given that more than 80% of those interviewed reported feeling anxious about getting infected, more than 60% were unemployed and fewer than 20% of households were food secure. This is contrary to a recent review assessing the psychological impact of the lockdown, which found that being quarantined resulted in a higher prevalence of psychological problems such as depression and anxiety, especially in those who were pregnant or had young children [45]. The review further suggests that while stressors including experiencing financial loss, fear of infection and stigma from others increased psychological problems during quarantine, the longer the duration of quarantine, the greater the psychological distress. Possible explanations for the low prevalence found in our study could be due to the short length of quarantine as data collection began approximately five weeks into the lockdown, and the consequences of the financial and other losses may not yet have been felt.

We found that the proportion of women experiencing psychological distress during the lockdown was higher than those who were distressed at their first clinic visit. While the lack of a control group makes it difficult to draw strong conclusions about the effect off the COVID-19 lockdown on symptoms of depression and anxiety, evidence from a longitudinal study in the same population [7] indicates that symptoms of depression detected early in pregnancy tend to abate during the course of pregnancy and the first year of the baby’s life. Our findings indicate the opposite, namely an increase in prevalence of psychological distress. As there were no other significant societal level events during this period that may have contributed to such an increase, we believe there is some support for the hypothesis that it was due to the COVID-19 lockdown. We did not observe a change in the number or proportion of women who felt suicidal. This is not out of keeping with other research; when examining studies reporting on suicidal thoughts and behaviour during pregnancy and the postpartum period, several studies have reported a decreased risk [46–48].

In LMIC, poverty is a well-documented risk factor for CMDs, especially during the perinatal period [49, 50]. The relationship is considered to be complex and bidirectional with several social issues interacting [51, 52]. Similar strong associations between food insecurity and CMDs have been consistently reported [4, 53, 54]. In a particularly low-resource setting in Cape Town, the odds of depression was five times greater in perinatal women who were food insecure compared to those who were food secure, while the odds of experiencing food insecurity was four times greater in women who were depressed compared to those who were not [4]. Our study found that being severely food insecure doubled the odds of CMDs during the COVID-19 lockdown, and that 80% of participants reported experiencing various levels of food insecurity. The high prevalence of food insecurity can be attributed to the COVID-19 lockdown. During April and May 2020, when South Africa was at Alert Level-4 and Level-5 of the lockdown, all non-essential services were halted. This resulted in high levels of unemployment, affecting the most vulnerable workers, who were low-skilled and less educated, the most [55]. Six weeks into the lockdown, Statistics South Africa, using an online survey, found that 4.3% of respondents reported experiencing hunger during the month prior to the lockdown, while 7% experienced hunger during the lockdown [56]. This is likely being under reported as those living in poverty and experiencing hunger would be unlikely to have access to a web-based survey. In our already vulnerable group of perinatal women living in low-resource settings in Cape Town, we found that more than 40% were severely food insecure. This translates to 40% of perinatal women living in households where they were eating fewer meals than needed, lacked the resources to acquire more food, went to sleep hungry or went a whole day and night without eating [41].

We found that the risk of CMDs was almost three times greater in women experiencing psychological or sexual abuse. However, we found quite a low prevalence of domestic abuse in our study compared to findings in similar populations [57, 58]. We found that 15% of participants reported experiencing psychological abuse, 14% reported experiencing physical abuse and less than 2% reported experiencing sexual abuse. Malan et al. [57], using the same 12-month recall period as in our study, reported that more than 40% of perinatal women experienced psychological abuse and 25% experienced sexual abuse. Schneider et al*.* [58] reported that 13.9% of pregnant women reported experiencing physical and/or sexual abuse. The lower prevalence in our study may be attributed to the telephonic data collection method used and the fact that the interviews were conducted during the lockdown period when the majority of South Africans were still confined to their homes. Participants may have under reported their experiences of abuse as their perpetrators were likely able to hear their responses to the interview questions.

Our study has a number of limitations. Selection bias may be present as more than half the women were not contactable. While levels of psychological distress before the lockdown were similar in those who were and were not contactable, it is possible that some women could not be contacted due to factors associated with socio-economic adversity, such as not being able to afford a cell phone. This study could therefore have under reported the level of unemployment and food insecurity, as well as the associated adverse outcomes such as CMDs and domestic violence. We did not have a control group, making it difficult to draw strong conclusions about the effect of the COVID-19 lockdown on depression and anxiety. We used only quantitative questionnaires which did not allow us to fully explore the perceived causes of CMDs, food insecurity and experiences of violence. We were only able to compare psychological distress as measured by a brief screening tool across the two time points, instead of using a validated tool such as the EPDS. In addition, the first screening was done at healthcare facilities by healthcare workers, while the second screening was telephonically administered by fieldworkers. The prevalence of CMDs could be under reported in the first screening, as patients may not have been willing to disclose personal information regarding their mood to healthcare workers whose primary role is perceived to be providing physical care rather than emotional support. Bland–Altman analysis has been used to account for the mean difference in measurement methods. We started data collection on the same day that the alcohol ban was lifted, which did not give us enough time to measure the impact of alcohol sales on domestic violence.

Further research is needed (1) to understand the coping mechanisms used by perinatal women to mitigate the stress of living in households with high levels of unemployment, food insecurity and domestic violence; (2) to investigate the effect that lifting the alcohol ban has had on domestic violence; and (3) to examine longer term trends in mental health, domestic abuse and food insecurity among perinatal women during the COVID-19 lockdown.

## Conclusion

This study provides evidence of the effect of the COVID-19 lockdown on the mental health status of perinatal women living in low-resource settings in Cape Town. The COVID-19 lockdown triggered high levels of unemployment and increased the prevalence of food insecurity, resulting in an increase in psychological distress being experienced during the lockdown, compared to before the lockdown. Our findings highlight how a crisis such as the COVID-19 lockdown amplified the psycho-social risk factors associated with CMDs in perinatal women.

## Supplementary Information

Below is the link to the electronic supplementary material.

Supplementary file1 (DOCX 21 KB)

## Data Availability

The dataset included for the analyses within this manuscript can be obtained on request from the corresponding author.
