# Supplementary material for: The relationship between common mental disorders (CMDs), food insecurity and domestic violence in pregnant women during the COVID-19 lockdown in Cape Town, South Africa
Source: Soc Psychiatry Psychiatr Epidemiol. 2021 Jul 19;57(1):37–46. doi: 10.1007/s00127-021-02140-7 (PMC8288830; doi:10.1007/s00127-021-02140-7)
Supplement: Supplementary file 1 — Supplementary file1 (DOCX 21 KB) [file 127_2021_2140_MOESM1_ESM.docx]

**Supplementary table:** Demographic characteristics of participants who were interviewed and participants who were lost to follow-up

|  | **Total**  **n=2149** | **Included in study**  **(n= 885; 41.2%)**  **n (%)** | **Lost to follow-up**  **(n=1264; 58.8%)**  **n (%)** | **Chi-square tests of independence** |
| --- | --- | --- | --- | --- |
| **Age** | | | | |
| 15-24 years | 824 | 314 (38.1) | 510 (61.9) | χ^2^(3) = 7.970; p=0.047 |
| 25-29 years | 517 | 215 (41.6) | 302 (58.4) |  |
| 30-35 years | 492 | 209 (42.5) | 283 (57.5) |  |
| >35 years | 189 | 92 (48.7) | 97 (51.3) |  |
| **Gravidit**y | | | | |
| 1^st^ pregnancy | 613 | 256 (41.8) | 357 (58.2) | χ^2^(2) = 0.795; p=0.672 |
| 2-4 pregnancies | 1354 | 559 (41.3) | 795 (58.7) |  |
| >4 pregnancies | 176 | 67 (38.1) | 109 (61.9) |  |
| **Pregnancy status** | | | | |
| Pregnant | 1628 | 688 (42.3) | 940 (57.7) | χ^2^(1) = 2.751; p=0.097 |
| Post-birth | 509 | 194 (38.1) | 315 (61.9) |  |
| **Mental health screening score** | | | | |
| Score=0 | 1062 | 472 (44.4) | 590 (55.6) | χ^2^(3) = 4.649; p=0.199 |
| Score=1 | 141 | 58 (41.1) | 83 (58.9) |  |
| Score=2 | 40 | 12 (30.0) | 28 (70.0) |  |
| Score=3 | 16 | 5 (31.2) | 11 (68.8) |  |
